# Supplementary material for: Molybdenum Disulfide Quantum Dots Prepared by Bipolar-Electrode Electrochemical Scissoring
Source: Nanomaterials (Basel). 2019 Jun 21;9(6):906. doi: 10.3390/nano9060906 (PMC6630415; doi:10.3390/nano9060906)
Supplement: Supplementary file 1 [file nanomaterials-09-00906-s001.pdf]

Supporting Information

Molybdenum Disulfide Quantum Dots Prepared by Bipolar-Electrode Electrochemical Scissoring

Yang Li [1], Xiaoxia Wang [1], Mengli Liu [1,2], Heng Luo [3], Lianwen Deng [3], Lei Huang [2], Shuang Wei [1], Congli Zhou [1], and Yuanhong Xu [1,2,*]

1 College of Materials Science and Engineering, Qingdao University, Qingdao 266071, China; liyang4875@outlook.com (Y.L.); wxx@qdu.edu.cn (X.W.); 15705420340@163.com (M.L.); 11181011018@stu.ouc.edu.cn (S.W.); 2017021070@qdu.edu.cn (C.Z.)

2 College of Life Sciences, Qingdao University, Qingdao 266071, China; lei_hl@126.com

3 College of Physics and Electronics; Institute of Super-Microstructure and Ultrafast Process in Advanced Materials; Central South University, Changsha 410083, China; luohengcsu@csu.edu.cn (H.L.); denglw@csu.edu.cn (L.D.)

* Correspondence: yhxu@qdu.edu.cn; Tel.: +86-532-83780128

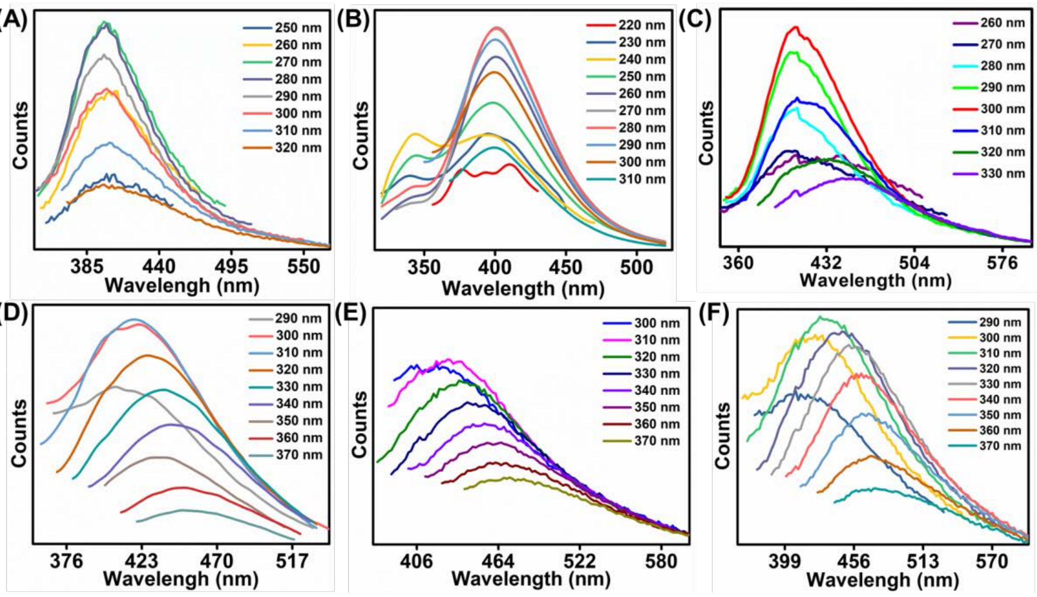

Figure S1. FL emission spectra with various excitation wavelengths of the supernatant under different peeling conditions. (**A**) 0.2 M NH_4F, 5 V, 20 h; (**B**) 0.2 M H_2SO_4, 5 V, 20 h; (**C**) 0.2 M PBS (pH = 7.4), 3 V, 20 h; (**D**) 0.2 M PBS (pH = 7.4), 7 V, 20 h; (**E**) 0.2 M PBS (pH = 7.4), 5 V, 10 h; (**F**) 0.2 M PBS (pH = 7.4), 5 V, 30 h.

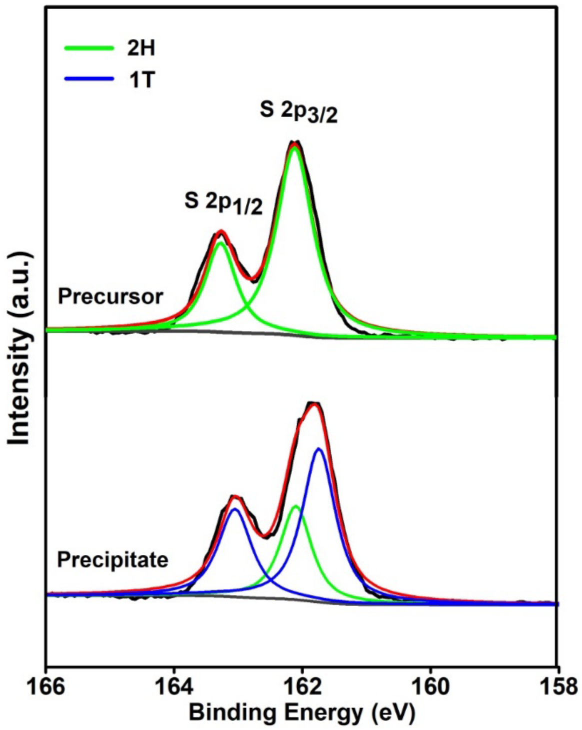

Figure S2. XPS spectra of the S 2p peak regions of MoS_2 precursor (above) and MoS_2 precipitate (below) samples.

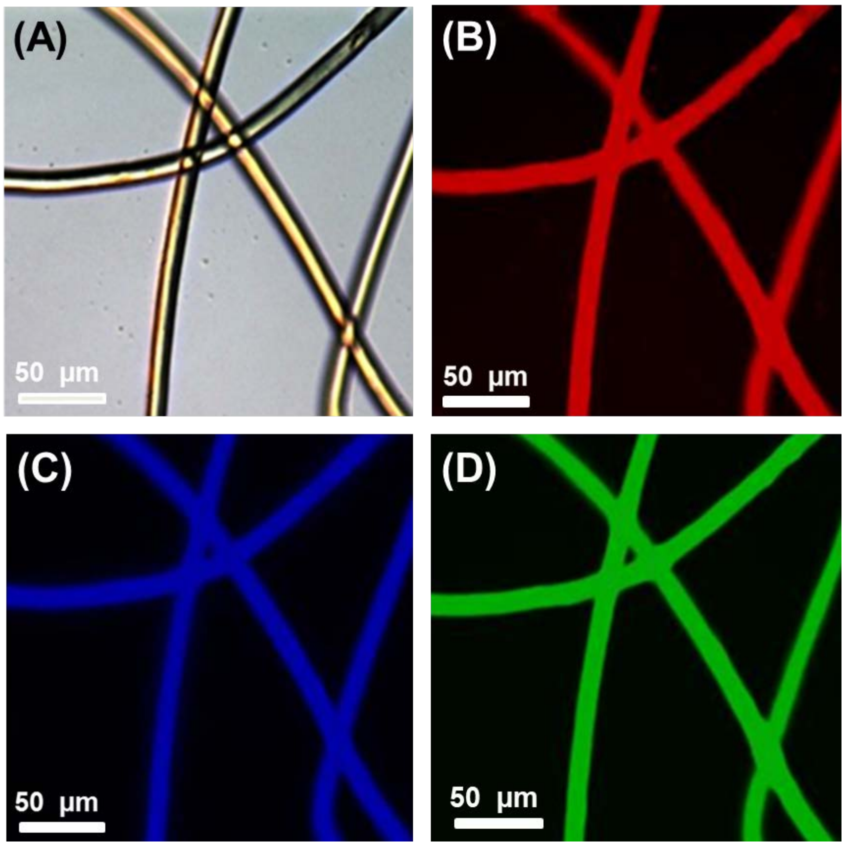

Figure S3. (**A**) Bright-field and (**B**–**D**) fluorescent images of cotton fibres stained with MoS_2 QDs. The fluorescent images were obtained at the excitation wavelengths of (B) 510–550 nm, (**C**) 330–385 nm, (**D**) 450–480 nm. Scale bar: 50 mm.

Table S1. To further elucidate the novelty of this work, comparison of previous works with this one.

Method	Precursor	Experimental Condition	Quantum Yield	Application	Advantage	Ref.
Ultrasonication	Natural Molybdenite	Ethylene glycol	-	-	Easy, efficient, cheap and environmentally benign preparation method; MoS_2 nanosheets maintaining the semiconducting properties; low toxicity.	43
Lithium (Li) intercalation	2H-MoS_2 powder	2.2 M n-butyl lithium solution in hexane	-	-	Effective and controllable preparation of luminescent monolayer MoS_2 QDs with a narrow size distribution.	44
Electrochemica l exfoliation	MoS_2 flakes (as electrode)	Lithium bis-(trifluorosulfon)imide (LiTFSI)	-	hydrogen evolution reaction	MoS_2 QDs with narrow size distribution; MoS_2 QDs exhibit excellent electrocatalytic activity towards hydrogen evolution reactions	13
Electro-Fenton reaction	MoS_2 crystalline powder	Sodium cholate, 0.05 M $FeSO_4$ (pH = 3)	-	-	Simple, cost-effective, efficient, and controllable method.	8
Hydrothermal method	Sodium molybdate and Cysteine	Water	2.6%	PL sensor for detection of TNP	Simple preparation method; Constructed a high sensitivity photoluminescence (PL) quenching sensor for detecting TNP.	24
Na intercalation reaction	Bulk MoS_2 (diameter of particles <2 μm)	Na	11%	fluorescent probe for long-term live cell tracing	Without using any toxic organic reagents during the preparation process; The as-prepared MoS_2 QDs were strongly fluorescent, highly photo-stable, low in cytotoxicity, and readily reactive to thiols.	45
Bipolar-elect-ro de (BPE) electrochemical method	MoS_2 powder (Mol. Wt. 160.07, purity 98.0%)	0.2 M PBS (pH = 7.4)	13.9%	MoS_2 QDs (fluorescent staining and cell imaging), byproduct (electromagnet ic wave absorber)	A new method for preparing MoS_2 QDs and MoS_2 electromagnetic wave absorbents; Without using any toxic organic reagents during the preparation process; High quantum yield; Simple	this wor k

Experimental Section

Quantum yield (QY) measurements: QY of the MoS_2 QDs was determined by previously established procedure [21]. Typically, quinine sulfate (literature quantum yield: 0.54) in H_2SO_4 (0.1 M) was chosen as a standard [26]. To minimize the re-absorption effects, the absorbance of the MoS_2 QDs dispersion and reference sample should be kept below 0.10 and 0.05 when excited at 310 nm, respectively. Quinine sulfate was dissolved in H_2SO_4 (0.1 M) while the MoS_2 QDs were dissolved in deionized water. The quantum yield of the MoS_2 QDs was calculated using the equation below [24]:

$$\Phi_X = \Phi_{ST}(\frac{Grad_X}{Grad_{ST}})(\frac{\eta_X^2}{\eta_{ST}^2}), \tag{1}$$

Where the subscripts ST and X refer to quinine sulfate and MoS_2 QDs, respectively, Φ represents the fluorescence QY. Grad stands for the gradient from the plot of integrated fluorescence intensity vs absorbance, and η is the refractive index of the corresponding solvent.

The intracellular uptake of MoS_2 QDs, bio-imaging and MTT assays: MTT assays were used to evaluate the MoS_2 QDs doses on the viability of the bamboo fibre cells. The cells were treated with

various concentrations of MoS_2 QDs (0, 50, 100, 150, 200, 250, 300 μg mL^{-1}) in fresh DMEM for 24 h. Treated cells were mixed with DMEM containing MTT (10 mL, 5 mg mL^{-1} in PBS solution) and further incubated at 5% CO_2, 37 °C for 4 h. Then the MTT containing medium was added to each well with 100 μL DMSO to solubilize the formazan crystals precipitate. The viability of untreated control cells was arbitrarily defined as 100%. Finally, the absorption at 490 nm of each well was measured by an EL808 ultramicroplate reader (Bio-TEK Instrument, Inc., Winooski, VT, USA). Bamboo fibre cells (106 cells per sample) were plated onto 35 mm glass chamber slides. The storage concentration of as-prepared MoS_2 QDs dispersion was about 300 μg mL^{-1}. MoS_2 QDs dispersion at the concentration of 60 μg mL^{-1} in DMEM was then freshly prepared and placed over the cells for 4 h at 37 °C. Subsequently, the cells were washed thoroughly three times with PBS to remove the free and physically absorbed MoS_2 QDs. Finally, the cellular images were taken by a Leica TCS SP2 confocal laser scanning microscope (CLSM) (Leica Microsystems Heidelberg GmbH, Germany) with an excitation wavelength of 360 nm from the Ar laser.
